# Kinases and protein motifs required for AZI1 plastid localization and trafficking during plant defense induction

**DOI:** 10.1111/tpj.15137

**Published:** 2021-02-20

**Authors:** Nicolás M. Cecchini, DeQuantarius J. Speed, Suruchi Roychoudhry, Jean T. Greenberg

**Affiliations:** ^1^ Department of Molecular Genetics and Cell Biology The University of Chicago 929 East 57th Street GCIS 524W Chicago IL 60637 USA; ^2^ Centro de Investigaciones en Química Biológica de Córdoba (CIQUIBIC‐CONICET) and Departamento de Química Biológica Ranwel Caputto Facultad de Ciencias Químicas Universidad Nacional de Córdoba Haya de la Torre y Medina Allende – Ciudad Universitaria Córdoba X5000HUA Argentina; ^3^ Centre for Plant Sciences University of Leeds Leeds LS2 9JT UK

**Keywords:** Systemic immunity, *Arabidopsis thaliana*, signal‐anchored proteins, defense priming, lipid transfer protein, AZI1

## Abstract

The proper subcellular localization of defense factors is an important part of the plant immune system. A key component for systemic resistance, lipid transfer protein (LTP)‐like AZI1, is needed for the systemic movement of the priming signal azelaic acid (AZA) and a pool of AZI1 exists at the site of AZA production, the plastid envelope. Moreover, after systemic defense‐triggering infections, the proportion of AZI1 localized to plastids increases. However, AZI1 does not possess a classical plastid transit peptide that can explain its localization. Instead, AZI1 uses a bipartite N‐terminal signature that allows for its plastid targeting. Furthermore, the kinases MPK3 and MPK6, associated with systemic immunity, promote the accumulation of AZI1 at plastids during priming induction. Our results indicate the existence of a mode of plastid targeting possibly related to defense responses.

## Significance Statement

In this work, we studied how AZI1, a key factor for plant systemic immunity, localizes to plastids. We show that AZI1 belongs to a unique class of signal‐anchored proteins and that the defense‐associated kinases MPK3/6 affect its targeting/trafficking, which in turn might determine the magnitude of systemic movement of defense signal(s) for resistance and priming induction.

## INTRODUCTION

Plants have an innate, non‐adaptive, immune system based on the ability to recognize non‐self‐molecules (Spoel and Dong, 2012). Pathogen recognition depends largely on two types of proteins: pattern recognition receptors that reside on the plasma membrane and perceive microbe‐associated molecular patterns (MAMPs; e.g. flg22 peptide, derived from bacterial flagellin) (Macho and Zipfel, 2014), and intracellular resistance proteins (R proteins), receptors that recognize specific effector proteins, or the effector‐induced alterations on their targets, that are injected by pathogenic microbes to promote virulence (Jones and Dangl, 2006; Cesari, 2018). After pathogen recognition, plants trigger local defense responses, such as reactive oxygen species accumulation, callose depositions and the activation of key signaling kinases, including MPK3 and MPK6 (MPK3/6) (Gómez‐Gómez and Boller, 2000; Chinchilla *et al*., 2006; Boller and Felix, 2009; Schwessinger *et al*., 2011). Additionally, pathogen infections can also induce long‐lasting and broad‐spectrum systemic resistance (Fu and Dong, 2013; Pieterse *et al*., 2014). Depending on the plant tissue involved in the initial recognition, different types of systemic resistance programs are established. Aerial tissues induce systemic acquired resistance (SAR) (Fu and Dong, 2013), whereas roots trigger the so‐called induced systemic resistance (ISR) that extends to the aerial tissue (Pieterse *et al*., 2014). In addition, when a MAMP(s) is/are perceived, it can also induce systemic resistance termed MAMP‐triggered SAR (mSAR) (Mishina and Zeier, 2007; Cecchini *et al*., 2015b).

Systemic immunity programs are typically characterized by an alert or “primed” state, which allows the plant to efficiently reactivate its defenses (more rapidly and/or strongly) against a subsequent pathogen attack (Jung *et al*., 2009; Parker, 2009; Conrath *et al*., 2015; Martinez‐Medina *et al*., 2016). For the establishment of systemic immunity after microbial recognition, the local production of one or more signal molecules capable of moving to distal tissues and priming defenses is needed. Several systemic signals have been described and many appear to be acting together during different systemic resistance programs (Park *et al*., 2007; Truman *et al*., 2007; Jung *et al*., 2009; Chanda *et al*., 2011; Chaturvedi *et al*., 2012; Návarová *et al*., 2012; Wittek *et al*., 2014; Chen *et al*., 2018). One of those signals is the lipid‐derived azelaic acid (AZA). AZA is locally generated in plastid envelopes and possibly thylakoid membranes and moves from local leaves to the systemic tissues (Ren *et al*., 2008; Jung *et al*., 2009; Zoeller *et al*., 2012; Yu *et al*., 2013; Gao *et al*., 2014; Cecchini *et al*., 2015b). AZA can induce a primed state when exogenously applied to aerial tissues (Jung *et al*., 2009; Cecchini *et al*., 2015b). A key component shared between SAR, ISR and mSAR programs and the associated priming, is the lipid transfer protein (LTP)‐like AZI1 (AZELAIC ACID INDUCED 1), and its close paralog EARLI1 (EARLY ARABIDOPSIS ALUMINUM INDUCED 1) (Jung *et al*., 2009; Cecchini *et al*., 2015b). AZI1 is required for the systemic movement of AZA specifically affecting systemic, but not local disease resistance (Cecchini *et al*., 2015b). Remarkably, AZI1 is also needed for the action of other proposed systemic defense signals glycerol‐3‐phosphate, dehydroabietinal and pinene‐monoterpenes (Chaturvedi *et al*., 2012; Yu *et al*., 2013; Riedlmeier *et al*., 2017). Proteins proposed to posttranslationally alter AZI1 are MPK3/6, which can phosphorylate AZI1 *in vitro* (Pitzschke *et al*., 2014); these kinases have prominent roles in SAR, ISR and defense priming induced by AZA (Beckers *et al*., 2009; Cecchini *et al*., 2019).

**Figure 1:**
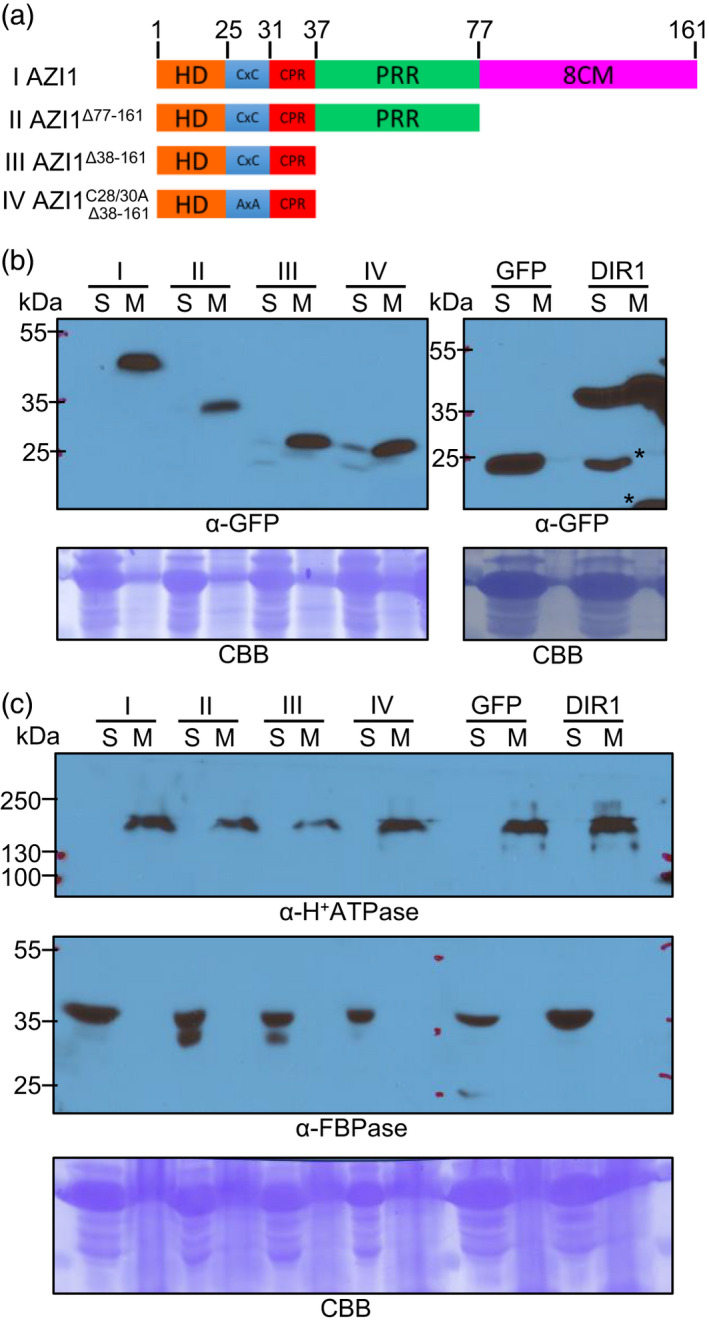
Membrane association of AZI1’s N‐terminal region. (a) Scheme of AZI1 deletion variants used in (b). Amino acid positions delimiting different AZI1 domains are shown in the upper part. Different variants are identified with roman numerals (left side). In AZI1 variant IV, both acylation sites were replaced by alanine (C28/30A). HD: hydrophobic domain/predicted signal peptide, similar to signal peptide, possible transmembrane domain; CxC: possible acylation sites; CPR: positively charged region; PRR: proline‐rich region; 8CM: lipid transfer domain (8‐cysteine motif). (b) Western blots of microsomal membrane (M) and soluble (S) protein fractions from *N. benthamiana* expressing GFP alone or fused to AZI1 variants (a) or DIR1:GFP. Bands were revealed using anti‐GFP antibody. Similar results were observed in three independent experiments. (c) Western blots of microsomal membrane (M) and soluble (S) protein fractions samples used in (b). Bands were revealed using anti‐H^+^ATPase and anti‐FBP antibodies as integral membrane protein and cytosolic markers, respectively. The blots in (b,c) stained with Coomassie blue (CBB) are presented to show loading. The asterisks indicate possible cleavage products.

AZI1 is a membrane protein and a pool of it, together with EARLI1, exists near the site of AZA production, the plastid outer envelope membrane (Zoeller *et al*., 2012; Cecchini *et al*., 2015b). AZI1 also localizes to the endoplasmic reticulum (ER), plasma membrane (PM) and plasmodesmata (Cecchini *et al*., 2015b; Lim *et al*., 2016). It was proposed that AZI1 forms part of membrane contact site complexes between plastids and ER membranes, allowing the non‐vesicular transport of AZA and possibly other non‐polar signals to systemic tissues (Cecchini *et al*., 2015b). After SAR‐triggering infections, AZI1/EARLI1 becomes highly enriched at plastid envelopes, which suggests that plastid targeting is critical for AZI1/EARLI1’s role in signaling (Cecchini *et al*., 2015b). However, AZI1 and EARLI1 do not possess a “classical” predicted or known signal sequence/motif that can explain their localization. AZI1 motifs can be divided into an amino terminal hydrophobic domain (HD, which is also a putative signal peptide (SP)), a central proline‐rich region (PRR) with unknown function, and a C‐terminal LTP domain (8 cysteine motif; 8CM) predicted to bind lipids (Figure 1a). Proteins like AZI1 that have a PRR plus an 8CM domain are considered “Hybrid Proline Rich Proteins” (HyPRPs) (Dvoráková *et al*., 2007). Previously, it was suggested that AZI1 employs an undescribed N‐terminal bipartite signal (SP+PRR) that drives its plastid targeting (Cecchini *et al*., 2015b).

Although many proteins that are localized to plastids show no recognizable signals, plastid targeting mechanisms for nuclear‐encoded proteins have been defined to some extent and can be divided into three groups: (1) targeted proteins with cleavable transit peptides or pre‐sequences that are recognized in the plastid envelope and then imported to the stroma, thylakoids or move back to inner or outer envelopes (Lee *et al*., 2017); (2) proteins with no cleavable signal where a transmembrane domain (TMD) acts as a targeting signal and anchors them to the outer envelope membrane (Kim and Hwang, 2013); (3) and the (less understood) plastidic β‐barrel proteins in which the secondary/tertiary structures constitute the organelle targeting region (Lee *et al*., 2014). In addition, TMD‐driven plastid proteins can be separated into tail‐anchored (C‐terminal region TMD) or signal anchored (N‐terminal region TMD) (Kim and Hwang, 2013). Signal‐anchored proteins are flanked by a charged positive region (CPR), usually containing at least three basic residues, important for the targeting as an ER import evading signal (Waizenegger *et al*., 2003; Lee *et al*., 2011). It was proposed that the degree of hydrophobicity of the TMD determines whether the region will anchor to plastid, mitochondria or ER membranes. Although there is a degree of overlap, signal‐anchored proteins in which the TMDs show a Wimley and White hydrophobicity score >0.4 mainly target the ER, whereas those with lower values mainly target plastids and/or mitochondria (Lee *et al*., 2011; Lee *et al*., 2014).

Here, we characterized AZI1 motifs in relation to their impact on subcellular targeting (with a focus on plastids) and identified pathogen defense components that modulate AZI1 localization. We report that specific features of the AZI1 amino terminus and the defense‐associated kinases MPK3/6 mediate AZI1‐plastid targeting and/or intracellular trafficking. Our results suggest the existence of a mechanism of plastid targeting and trafficking that is active during defense responses against pathogens.

## RESULTS

### AZI1 is a signal‐anchored protein

The TargetP algorithm (Emanuelsson *et al*., 2007) predicts that AZI1’s hydrophobic amino terminus is a signal peptide. However, since AZI1 strongly associates with membranes (Cecchini *et al*., 2015b), this domain might be a non‐cleavable TMD anchor, as has been shown for many signal‐anchored proteins (Figure 1a I) (Jayasinghe *et al*., 2001; Kim and Hwang, 2013). In support of this idea, amino acids 31–37 (KPSPKPK) in AZI1’s amino terminus constitute a charged protein region (CPR) that is characteristic of signal‐anchored proteins (Figure 1a I).

To further analyze the possibility that AZI1’s N‐terminal region (AZI1^Δ38‐161^ containing the HD+CPR) functions as a signal anchor, we tested if it was sufficient to confer membrane association. We generated a construct where AZI1^Δ38‐161^ was fused to a GFP construct (Figure 1a III) and analyzed its localization by fractionation. AZI1 fused to GFP was previously shown to retain function (Cecchini *et al*., 2015b). Microsomal and soluble fractions were obtained from agroinfiltrated *Nicotiana benthamiana* leaves and examined by immunoblot. A large pool of the AZI1^Δ38‐161^:GFP fusion protein was found in the microsomal fraction (M), with only traces present in the soluble fraction (S) (Figure 1b III). Because AZI1 also possesses two possible acylation sites after the HD (C28/C30; CSS‐Palm 2.0; (Ren *et al*., 2008)) that could relate to its microsomal localization, we repeated the analysis using a second construct with these residues mutated (cysteines to alanines, CxC→AxA) (Figure 1a IV). Figure 1(b IV) shows that this variant also largely partitioned with the microsomal fraction, indicating that these sites do not significantly affect membrane localization. As previously shown, full length AZI1 or AZI1 without the LTP domain (8CM) also localized to microsomal fractions (Figure 1b I and II) (Cecchini *et al*., 2015b). As controls, the soluble GFP and the soluble/microsomal DIR1:GFP constructs showed the expected fractionation patterns indicating that we can detect transiently expressed proteins located to the soluble and membrane fractions of *N. benthamiana* (Figure 1b, right panel) (Cecchini *et al*., 2015b). In addition, the distribution of markers for microsomal and soluble fractions, H^+^ATPase and FBPase, respectively, indicated that the fractionation worked as anticipated (Figure 1c).

Together, these results strongly suggest that the N‐terminal 37 amino acids that include AZI1’s putative SP (the HD region in Figure 1) are sufficient to confer membrane anchoring, indicating that AZI1 is a signal‐anchored protein.

### The PRR and TMD are required for the normal pattern of AZI1 plastid envelope targeting

**Figure 2:**
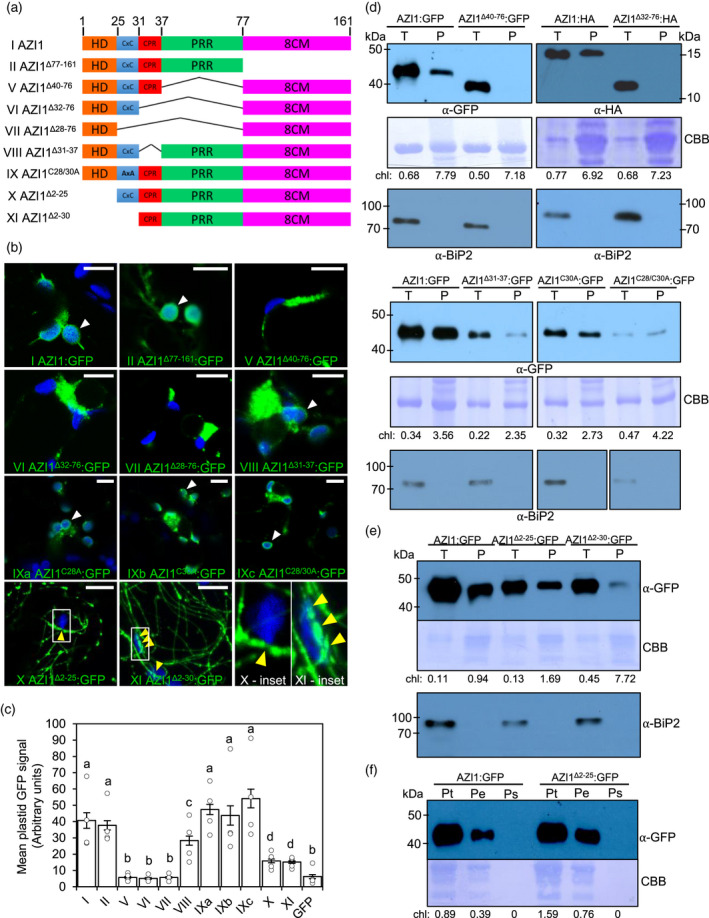
AZI1 protein regions required for its plastid targeting. (a) Scheme of AZI1 deletion and mutant variants used for the localization pattern analysis in (b) and subcellular fractionation in (d). Amino acids positions delimiting AZI1 domains are shown in the upper part. Different variants are identified with roman numerals (left). See Figure 1(a) for the definitions of each region. (b) Laser scanning confocal microscopy micrographs showing localization of GFP‐tagged AZI1 variants (a) expressed in *N. benthamiana*. AZI1 variant IX includes different AZI1 versions where acylation sites were replaced by alanine (version IXa:C28A, IXb:C30A, IXc: C28/30A). AZI1 variants X and XI inset panel (at bottom‐right position) shows a close up of the filamentous signal overlapping (“touching”) with plastid autofluorescence. White arrowheads indicate GFP fluorescence in plastid envelopes (note the rings of GFP signal in green that surround autofluorescent plastids in blue in the micrographs). Yellow arrowheads indicate filamentous GFP signal overlapping plastid autofluorescence. Bar = 10 µm. (c) Quantification of mean GFP signal intensity of AZI1 and its variant constructs within the plastids. The GFP signal intensity from 20 to 25 plastids was quantified for each construct, from 4 to 5 independent imaging experiments. The bars represent standard errors of means of these values. Individual data points are shown on the bar chart as scatter‐dots. (d–f) Western blots of plastid (P) and total (T) fractions and total (Pt), envelope (Pe) and soluble (Ps) plastid subfractions from *N. benthamiana* expressing GFP‐ or HA‐tagged AZI1 or AZI1 deletion and mutation variants. Bands were revealed using anti‐GFP or anti‐HA antibody as indicated. Asterisks indicate unspecific bands. The blot stained with Coomassie blue (CBB) is presented to show loading. 7–10 ug of protein were loaded on blots probed with anti‐GFP antibody and 30 ug of protein were loaded on the blot probed with anti‐HA antibody. Chlorophyll amount (µg) is shown for each fraction to indicate the plastid enrichment. Similar results were observed in two or more independent experiments. Western blots of the same total and plastid extracts were also probed with anti‐BiP2 to assess the level of ER contamination in plastid fractions. For the blots containing variants VIII, IXb, and IXc, the lanes containing the size marker between variants were cropped from the images. For the panels displaying variants VIII, IXb, and IXc, the anti‐GFP and anti‐BiP2 blots were yielded from separate SDS‐PAGE gels.

Most signal‐anchored proteins targeted to plastid envelopes (or mitochondria) have an N‐terminal TMD Wimley and White hydrophobicity score below 0.4 (Lee *et al*., 2011). In contrast, AZI1’s HD displays a 0.58 score, indicative of an ER membrane resident signal‐anchored protein (MPEx software 3.2, amino acids 8–26, http://blanco.biomol.uci.edu/mpex/index.html; Jayasinghe *et al*., 2001; Kim and Hwang, 2013)). Thus, to determine whether other domain/motifs are required for AZI1 plastid outer envelope membrane targeting, we generated AZI1 variants for each one (Figure 2a) and fused them to GFP or an HA tag.

To test the subcellular localization of AZI1‐GFP variants, we imaged agrotransformed *N. benthamiana* leaves by confocal microscopy. The deletions of the PRR motif (AAs 40‐76), or CPR+PRR (AAs 32‐76) and CxC+CPR+PRR (AAs 28‐76) regions, abolished AZI1’s plastid targeting (Figure 2b, compare I and II to V, VI and VII). Quantitation of the GFP fluorescence showed a significant decrease in GFP signal localized to plastids upon deletion of these regions (Figure 2c I, II, V, VI, and VII). Consistent with our previous work (Cecchini *et al*, 2015b), fractionation confirmed the loss of plastid targeting for PRR deletion variants V and VI; quantitation of chlorophyll and immunoblot analysis of BiP2 indicates that chloroplast enrichment was successful without significant ER contamination (Figure 2d). In contrast, plastid localization was largely retained in AZI1 variants upon the loss of the CPR region or putative acylation sites (Figure 2b–d I, VIII, and IX). Though non‐essential, the CPR may weakly affect plastid targeting (Figure 2c VIII). We also performed microscopy on two AZI1 versions fused to GFP where the HD or the HD plus the following acylation sites were deleted (Figure 2a, X and XI). Neither variant displayed AZI1’s characteristic ring‐like localization around plastids or clear ER or plasma membrane localization sites (Figure 2b X and XI). Instead, both the AZI1^Δ2‐25^:GFP (X) and AZI1^Δ2‐30^:GFP (XI) fluorescence signal displayed a filamentous pattern as if associated with actin filaments or microtubules (Figure 2b, X and XI). Surprisingly, even though neither variant displayed AZI1’s ring‐like localization around plastids, fractionation revealed that both variants X and XI associate with plastids (Figure 2e). Further partitioning of plastids into membrane and soluble fractions indicates that variant X is plastid‐membrane associated (Figure 2f). Close inspection of the confocal micrographs supports the fractionation results, as there were points of contact between GFP filaments and plastids (Figure 2b, X and XI yellow arrowheads, and X and XI insets at bottom‐right panel). This was also observed in the GFP fluorescence quantitation when compared to control GFP (Figure 2c X and XI). We noted that variants X and XI were similar in apparent mass to AZI1 (Figure 2e). It is possible that the higher percentage of proline residues in AZI1 variants relative to AZI1 causes anomalous migration patterns during SDS‐PAGE similar to what has been previously described for other proline‐rich proteins (Hames, 1998). Alternatively, higher than expected migration may be due to extra posttranslational modifications related to the formation into filaments of variants X and XI.

These results corroborate our earlier observations about the importance of the PRR for plastid targeting (Cecchini *et al*., 2015b). Furthermore, they show that the HD/TMD is required for the normal ring‐like pattern of plastid membrane association. Together, they indicate that the motifs required for full AZI1 plastid outer envelope membrane targeting are the HD/TMD plus the PRR (Table S1), suggesting a bipartite signal‐anchored protein targeting mechanism.

### Loss of the TMD causes AZI1 to stably associate with microtubules

**Figure 3:**
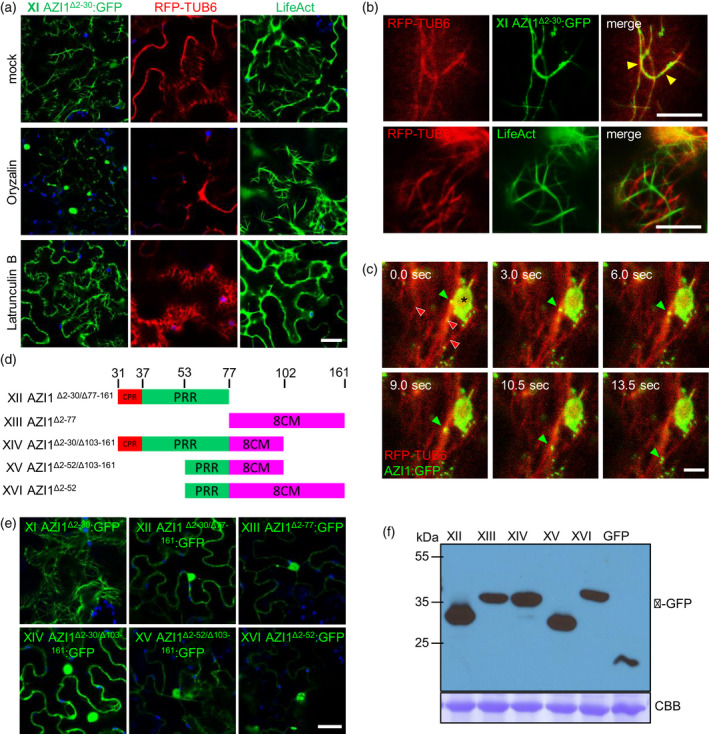
Co‐localization of AZI1 variants with the microtubule network. (a–c,e) Laser scanning confocal microscopy micrographs showing localization of GFP‐tagged AZI1 variants, microtubule marker (RFP:TUB6) and filamentous actin marker (LifeAct:GFP) in *N. benthamiana*. (a) Micrographs showing the effect of actin (Latrunculine B, LatB) and microtubule (oryzalin, Ozn) inhibitors or mock treatments on AZI1 variant XI:GFP, RFP:TUB6 or LifeAct:GFP localization patterns. (b) RFP:TUB6 is co‐expressed with AZI1 variant XI:GFP or LifeAct. (c) Time series micrographs showing dynamic localization of AZI1:GFP in vesicle‐like structures (green arrowhead) moving on microtubule bundle (red arrowhead; microtubule marker; RFP:TUB6). Asterisk indicates a plastid. sec, seconds. Micrographs show GFP (green), RFP (red) and plastid autofluorescence (blue). In (a,e) Bar = 20 µm, (c) Bar = 5 µm, and (b) upper panel Bar = 50 µm and middle and bottom panel Bar = 10 µm. (d) Scheme of AZI1 variants used in (e,f). Amino acid positions delimiting AZI1 domains are shown in the upper part. Different variants are identified with roman numerals (left). (f) Western blots of total protein from *N. benthamiana* expressing GFP alone or fused to AZI1 variants used in (e). Bands were revealed using anti‐GFP antibody. The blot stained with Coomassie blue (CBB) is presented to show loading.

The fluorescence patterns of AZI1‐GFP variants that lack the TMD showed strong similarities to microtubules (MTs) and/or actin filaments (AFs) (Figure 2b X and XI; Kang *et al*., 2014; Kumar *et al*., 2018). Therefore, we investigated the effects of Oryzalin (Ozn) and Latrunculin B (LatB), MT and AF inhibitors, respectively, on the AZI1^Δ2‐30^:GFP filamentous pattern. As Figure 3(a) (left panel) shows, Ozn but not LatB treatment vastly reduced AZI1^Δ2‐30^:GFP filaments compared with mock treatment. The effectiveness controls for the inhibitors, on MT‐targeted (RFP‐TUB6) and actin‐targeted (LifeAct) markers, showed the expected results (Figure 3a, middle and right panels). Moreover, when we co‐expressed AZI1^Δ2‐30^:GFP together with RFP‐TUB6, we observed a robust co‐localization between GFP and RFP signals (Figure 3b, upper panels). No obvious co‐localization was observed between RFP‐TUB6 and LifeAct markers (Figure 3b, bottom panels). Importantly, live imaging of AZI1:GFP together with RFP‐TUB6 showed that full length AZI1 in vesicle‐like structures moved in close association with MT bundles (Figure 3c and Movie S1). The proportion of AZI1:GFP colocalizing with RFP‐TUB6 represents ~16% of the total signal compared with a ~3% found in control GFP (Figure S1a). Thus, loss of the TMD anchor induced AZI1 to (more) stably associate with MTs network, suggesting that there is a close relationship between AZI1 and cell cytoskeleton. We considered the possibility that the association of AZI1^Δ2‐25^:GFP and AZI1^Δ2‐30^:GFP with discrete contact points on plastids (Figure 2b,e) might be due to the accumulation of these AZI1 variants along the MTs. However, tubulin was absent from plastid fractions, suggesting that these variants associate with plastids without stable/strong MT associations (Figure S1b).

Next, to analyze which of the AZI1 motifs/regions are required for AZI1^Δ2‐25/2‐30^:GFP association with MTs, we generated several deletion constructs for AZI1. Partial deletions fused to GFP were transiently expressed in *N. benthamiana* to assess the localization patterns (Figure 3d). Interestingly, none of the deletions generated showed a filamentous pattern comparable with AZI1^Δ2‐30^:GFP (Figure 3e), and instead, the constructs localized to the ER/cytoplasm and nuclei. Western blot analysis indicated that the GFP was not cleaved from the expressed fusion proteins (Figure 3f).

These results suggest that AZI1’s PRR and 8CM regions are required together for the direct or indirect association with MTs.

### Microtubules are dispensable for flg22‐induced enrichment of AZI1 to plastids

To dissect which cellular components are needed for AZI1 targeting, we began by identifying a defined, strong defense‐inducing stimulus other than pathogen infection (Cecchini *et al*., 2015b) that might cause enrichment of AZI1 to plastids. Since AZI1 and its paralogue EARLI1 are also needed for mSAR induction, we measured AZI1/EARLI1 levels in total and plastid extracts after flg22 treatment.

**Figure 4:**
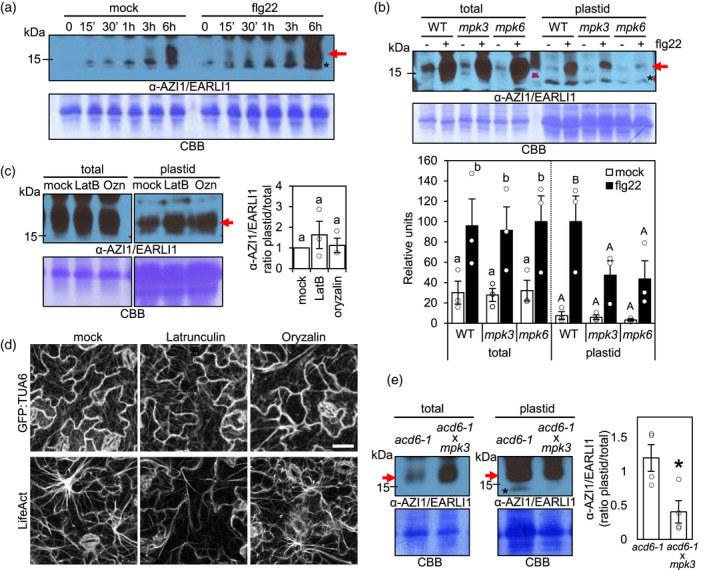
Impact of MAP kinase mutations and cytoskeleton inhibitors on AZI1/EARLI plastid targeting during defense signaling. (a) Western blot of total extracts from WT Col‐0 Arabidopsis leaves to test AZI1–EARLI1 protein levels at different times post infiltration with 1 µm flg22 or water (mock). (b) AZI1–EARLI1 levels in total and plastid fractions from *Arabidopsis* leaves of WT Col‐0, *mpk3,* and *mpk6* plants 12 h post treatment with 1 µm flg22 (+) or water (mock, −). Shown in the graph is quantitation of AZI1–EARLI1 levels relative to the total protein content in each Coomassie blue membrane lane, as quantified by densitometry. The highest value in total or plastid fractions was set to 100 in the relative units. (c) AZI1–EARLI1 protein levels in total and plastid fraction from Arabidopsis WT Col‐0 leaves 6 h post treatment with 1 µm flg22 and 3 h post‐infiltration with inhibitors of actin filaments (Latrunculine B, LatB), microtubules (oryzalin, Ozn) or mock. Right graph: ratio of AZI1/EARLI1 in plastids versus total in different treatments, as quantified by densitometry. Western blot panels separated with vertical line belong to the same blot. (d) Actin and microtubule cytoskeleton changes in *Arabidopsis* after inhibitor treatments. Col/Lifeact‐GFP and Col‐*gl1*/GFP‐TAU6 transgenic plants expressing actin or microtubule markers, respectively, were imaged by laser‐scanning confocal microscopy at 6 h post treatment with 1 µm flg22 and 3 h post‐infiltration with inhibitors of actin filaments (Latrunculine B), microtubules (oryzalin) or mock. The representative micrographs shown are Z‐series maximum intensity projections. Micrographs show GFP signal (greyscale). Bar = 20µm. (e) AZI1–EARLI1 protein levels in total and plastid fractions from untreated *Arabidopsis* leaves of *acd6‐1* and *acd6‐1mpk3‐1* mutant plants. Right graph: plastid to total ratio of AZI1–EARLI1 levels relative to the total protein content in each Coomassie blue membrane lane, as quantified by densitometry. The averages ± SE from three (b,c) or four (e) independent experiments are shown. In (b,c), different letters (lower or uppercase) indicate statistically significant differences (*P* < 0.01, analysis of variance (anova), SNK test (b) or Tukey test (c)). The dotted line indicates that the total and plastid fractions were analyzed independently in (b). In the graph in (e), the asterisk indicates statistically significant differences determined by *t*‐test (*P < 0.05, *n* = 4). In (b–d) the individual data points are shown on the bar chart as scatter‐dots. In (a–c,e) the blots stained with Coomassie blue (CBB) are presented to show loading. Bands were revealed using anti‐AZI1/EARLI1 polyclonal serum. Red arrow indicates the AZI1/EARLI1 monomer band. Asterisks indicate unspecific bands.

We infiltrated *Arabidopsis* WT plants with flg22 or H_2_O (mock) and analyzed the native AZI1/EARLI1 levels in total extracts at different times post‐treatment by Western blot. Figure 4(a) shows that between 3–6 h post treatment (hpt), the total amount of AZI1 greatly increased in response to flg22 (+) compared to mock (−). Local AZI1/EARLI1 induction by flg22 treatment was also pronounced at 12 hpt (Figure 4b, total – WT plants). To determine if this AZI1 increase translated to higher levels in plastids, we treated *Arabidopsis* with flg22 and analyzed the levels of AZI1/EARLI1 in total and plastid fractions at 6 and 12 hpt (Figure 4b; Figure S2a, WT plants). The amount of plastid‐localized AZI1/EARLI1 increased in flg22‐treated samples compared to mock; at 12 hpt there was a greater fold increase in the amount of AZI1/EARLI1 targeted to plastids (~10×) relative to the fold increase in the total extract (~3×). Thus, flg22 MAMP treatment strongly induces AZI1/EARLI1 protein levels and increases their relative enrichment in the plastid fraction.

Considering the above results and the role of cytoskeletal dynamics for protein trafficking, we next studied the importance of the cytoskeleton for AZI1 targeting to plastids during MAMP stimulation. We quantified AZI1/EARLI1 protein levels in *Arabidopsis* WT Col‐0 total and plastid fractions 6 hpt with flg22 and 3 h post‐infiltration with or without inhibitors for microtubules (Ozn) and actin filaments (LatB), respectively. This set up allowed us to make the inhibitor treatments before the earliest robust increase of AZI1 in plastids (Figure S2a; Figure 4a). As shown in Figure 4(c), Ozn or LatB treatments did not affect the plastid AZI1 levels compared to mock. As efficacy controls for the inhibitor treatments, we used the same treatment conditions to study transgenic plants expressing Col/Lifeact‐GFP and Col‐*gl1*/GFP‐TUA6, actin or microtubules markers, respectively. Both inhibitor treatments strongly disrupted the respective AF and MT cytoskeletons (Figure 4d).

These results suggest that although AZI1 can traffic in close association with MTs, disruption of the cytoskeleton does not significantly impact AZI1 targeting to plastids.

### MPK3 and MPK6 enhance AZI1/EARLI1 plastid targeting during defense induction

AZI1’s PRR is a proposed phosphorylation target for MPK3 and possibly MPK6 (Pitzschke *et al*., 2014), two key signaling factors associated with biotic and abiotic stress (Rodriguez *et al*., 2010). Moreover, we recently showed that these kinases are also needed for underground AZA‐induced priming (Cecchini *et al*., 2019). Since the PRR is required for plastid targeting (Figure 2), we analyzed if MPK3/6 affect plastid localization of AZI1/EARLI1 proteins.

We treated WT Col‐0, *mpk3* and *mpk6* mutant plants with flg22 or H_2_O (mock) and analyzed the levels of native AZI1/EARLI1 in plastids fractions compared to total extracts (Figure 4b; Figure S2a). Compared to WT, the *mpk3* and *mpk6* plants displayed decreased levels of AZI1/EARLI1 specifically in plastid fractions 12 h after flg22 treatment, but not in mock‐treated plastid fractions or any total extracts (Figure 4b). In addition, we also observed a reduction of AZI1/EARLI1 in *mpk6* plastids fraction at 6 hpt (Figure S2a). Immunoblots with phospho‐antibody show that MPK3/4/6 were still active at both 6 and 12 h post flg22 treatment compared with mock treated plants (Figure S2b).

Together, these results strongly suggest that both MPK3 and MPK6 are required for robust AZI1 plastid enrichment during flg22‐MAMP defense induction. Supporting this idea, *acd6‐1*, a plant with constitutively active MPK3 and pattern receptor‐mediated immunity (Tateda *et al*., 2014), showed a reduced proportion of AZI1/EARLI1 in the plastid fraction relative to the total extract when an *mpk3* mutation was present (Figure 4e). The lower total levels of AZI1 in *acd6‐1* versus *acd6‐1mpk3* may be due to an altered balance of signaling molecules/hormones including salicylic acid, which is affected by both mutations (Vanacker *et al*, 2001; Zhang *et al*., 2014).

### MPK3 and MPK6 are required for mSAR induction in Arabidopsis

**Figure 5:**
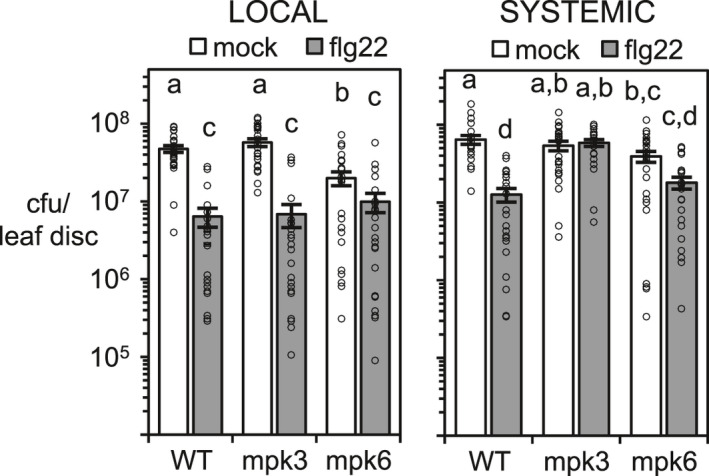
flg22 local and systemic disease resistance induction in Arabidopsis WT Col‐0, *mpk3* and *mpk6* plants. Growth of the virulent bacteria *Pma*DG3 on plants 3 days post infection (OD_600_ = 0.0003). *Pma*DG3 was infiltrated in local or distal leaves after 2 days of the local leaf treatment with water (mock) or 200 ηm flg22. The average of cfu per leaf disc ± standard error from three independent experiments (each one with eight biological replicates) is shown. Graph *y* axes are in log_10_ scale. Different letters show significant differences between treatments and/or mutants (*P* < 0.01, analysis of variance (anova), Tukey test). Individual data points are shown on the bar chart as scatter‐dots.

Considering the requirement of AZI1 and EARLI1 for mSAR (Cecchini *et al*., 2015b), we next analyzed if MPK3 and MPK6 are also needed for this systemic defense program. We pretreated by infiltrating three lower leaves of WT Col‐0, *mpk3* and *mpk6* plants with flg22 or H_2_O (mock), and 2 days later infected plants with the virulent *Pseudomonas* strain *Pma*DG3 in the same (local) or systemic leaves. Both MPK3 and MPK6 were required for systemic resistance induced by flg22, as judged by the growth of *Pma*DG3 (Figure 5, right graph). *mpk3* was completely mSAR defective and *mpk6* displayed a weak and statistically insignificant induction. As previously reported, local resistance after flg22 treatment was not affected in either *mkp3* or *mpk6* single mutant plants compared to WT plants (Figure 5, left graph) (Su *et al*., 2017). However, mock‐treated *mpk6* plants showed modestly increased resistance to *Pma*DG3 relative to the other genotypes tested.

These data indicate MPK3/6 are required for mSAR induction, possibly related to their effect on AZI1/EARLI’s plastid targeting. Because MPK3 and MPK6 are also required for SAR and ISR (Beckers *et al*., 2009; Cecchini *et al*., 2019), our data strongly indicate a key role for these kinases in plant systemic resistance programs.

### AZI1, MPK3 and MPK6 co‐localize at sites of plastid–ER contacts

It was previously suggested that MPK3 interacts with AZI1 and forms protein complexes near the cell boundary (Pitzschke *et al*., 2014). To get deeper insight into the subcellular localization and possible complex formation between AZI1 and MPK3/6, we analyzed their co‐localization by confocal microscopy.

**Figure 6:**
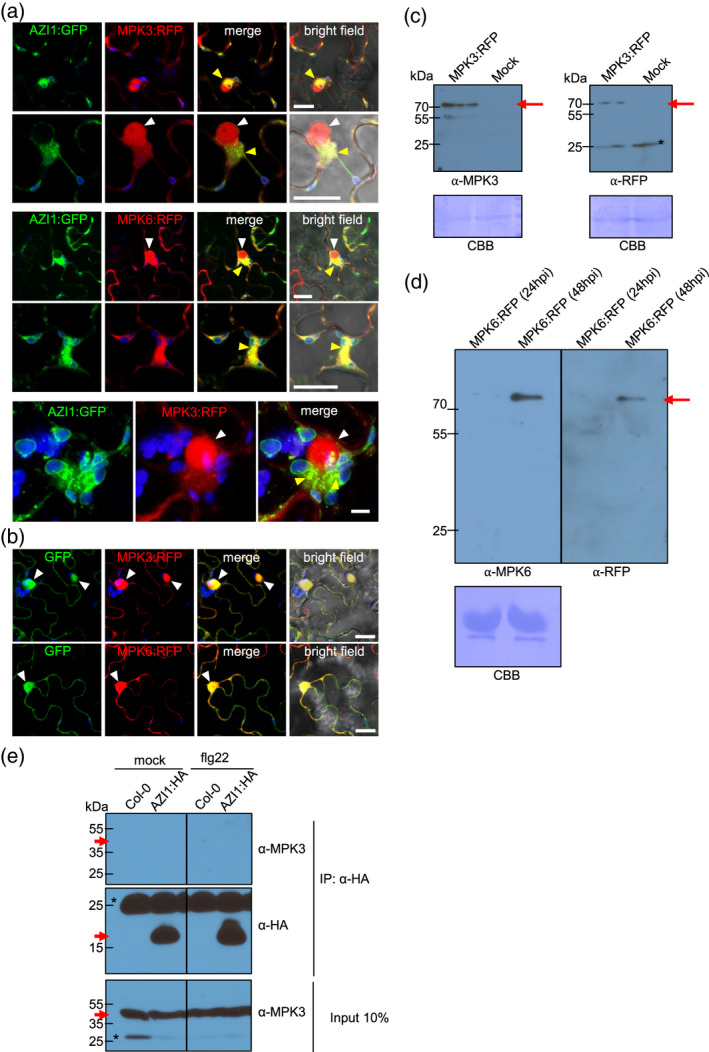
AZI1 and MPK3/6 co‐localization and complexes. (a,b) Laser scanning confocal microscopy micrographs showing localization of co‐expressed AZI1:GFP or control‐GFP and MPK3 and MPK6 RFP‐tagged (MPK3:RFP, MPK6:RFP) in *N. benthamiana*. Arrowheads indicate GFP and RFP fluorescence co‐localization. Yellow arrowheads: co‐localization at perinuclear ER and at plastids/stromule–ER contact sites; white arrowheads: nuclei. In (a) bottom panels: Z‐series maximum intensity projection showing close up view of AZI1:GFP and MPK3:RFP co‐localization. Micrographs show GFP (green), RFP (red) and plastids autofluorescence (blue). In (a) upper and middle panels and (b) Bars = 20 µm; (a) bottom panels Bar = 5 µm. (c,d) Western blot of total extracts from agrotransformed *N. benthamiana* tissues expressing MPK3:RFP (c) or MPK6:RFP (d). Red arrows indicate full length MPK3:RFP or MPK6:RFP fusion proteins. Bands were revealed using anti‐RFP, anti‐MPK3 or anti‐MPK6 antibodies as indicated. The blots stained with Coomassie blue (CBB) are presented to show loading. Asterisks indicate unspecific bands. In (d) the same blot was probed first with anti‐RFP and then reprobed with anti‐MPK3. In both cases, the blot was secondarily probed with anti‐rabbit. (e) Immunoprecipitation of AZI1 to test for association with MPK3 in Arabidopsis. WT Col‐0 and transgenic plants overexpressing AZI1:HA (line A6) were sprayed with Dex 30 µm in 0.1% Tween 20 solution. After 1 day, plants were infiltrated with 1 µm flg22 or water (mock) and 6 h later samples were harvested. Complexes were immunoprecipitated with HA antibody. Input (bottom panels) and immunoprecipitation (middle and upper panels) protein samples were analyzed by Western blot using the indicated antibodies. Western blot panels separated with vertical line belong to the same blot. Red arrows indicate the expected size band. Asterisks indicate unspecific bands. Similar results were observed in two independent experiments.

We co‐expressed MPK3:RFP or MPK6:RFP together with AZI1:GFP by agro‐transformation of *N. benthamiana* leaves. As shown in Figure 6(a), both MPK3:RFP and MPK6:RFP localized to the nucleus in a spherical pattern and partially co‐localized with AZI1:GFP at perinuclear ER and plastid/stromule–ER contact sites. Some co‐localization signals are likely to be plastid–ER and/or stromule–ER contact sites (Figure 6a; bottom panels) (Cecchini *et al*., 2015b). No co‐localization was found between control soluble GFP and MPK3:RFP or MPK6:RFP at those places (Figure 6b). As expected, control GFP and MPKs co‐localize at nuclei and cytoplasm. Immunoblots to detect MPK3 and MPK6 fusion proteins established that free RFP was not detectable (Figure 6c,d).

Next, we assessed putative interactions between AZI1 and MPK3 during defense induction. Immunoprecipitation (IP) assays were implemented by using *Arabidopsis* that expressed functional HA‐tagged AZI1 previously shown to complement *azi1* (Cecchini *et al*., 2015b). AZI1:HA‐expressing plants treated with flg22 or H_2_O (mock) for 6 hpt were used for immunoprecipitation with an anti‐HA matrix. MPK3 did not co‐precipitate with AZI1:HA in either mock‐ or flg22‐treated samples (Figure 6e).

Altogether, these results indicate that although they partially co‐localize, AZI1 does not form stable complexes with MPK3 in *Arabidopsis* under our assay conditions.

## DISCUSSION

Not much information exists about the subcellular targeting/trafficking regulation of immune components (Khaled *et al*., 2015; Wang *et al*., 2016; Gu *et al*., 2017). AZI1, a key factor for the plant systemic defenses, shows dynamic levels in plastid envelopes (Jung *et al*., 2009; Cecchini *et al*., 2015b). Interestingly, AZI1 does not have a recognizable “classic” plastid targeting signal. Here, we discovered and characterized a N‐terminal bipartite signature that explains AZI1’s subcellular location: a predicted signal peptide followed by a PRR (Cecchini *et al*., 2015b). AZI1’s putative signal peptide is a non‐cleavable TMD that anchors the protein to membranes, placing this LTP‐like protein as a signal‐anchored protein. In addition, unlike what was found for other signal‐anchored proteins, the presence of a PRR after the TMD (and not the TMD hydrophobicity (Kim and Hwang, 2013)) is required for plastid targeting. Together, our data establishe AZI1 as belonging to a previously undescribed class of signal‐anchored proteins. Remarkably, the defense‐associated kinases MPK3 and MPK6 are required for the increased targeting of AZI1 to plastids in response to flg22. Moreover, our results suggest that AZI1 utilizes the MT network for intracellular trafficking. Therefore, we propose that after pathogen or MAMP recognition, MPK3/6 are locally stimulated and, by acting on its N‐terminal bipartite signal, enhance AZI1 plastid targeting. This in turn could determine how much systemic defense priming ensues.

### AZI1 is a variant of signal‐anchored proteins

AZI1 is a signal‐anchored protein targeted to plastids. This finding explains our previous work showing that AZI1 is present in microsomal fractions (Cecchini *et al*., 2015b). Supporting this, other reports showed no cleavage of the AZI1 putative signal peptide (Zhang and Schläppi, 2007; Pitzschke *et al*., 2016). Because traces of AZI1 TMD:GFP were detected in the soluble fraction (AZI1^Δ38‐161^ and AZI1^C28/30A Δ38‐161^), we cannot completely rule out that a very small amount of AZI1 is capable of entering the secretory pathway. In agreement with this possibility, using protoplasts and root exudates, the localization of a small proportion of AZI1 was consistent with its being secreted to plant cell walls (Zhang and Schläppi, 2007; Pitzschke *et al*., 2016). Alternatively, soluble traces of AZI1 TMD:GFP in our experiments might be contamination from the microsomal extraction fractionation.

Most signal‐anchored proteins targeted to plastids have relatively low TMD hydrophobicity indices (Lee *et al*., 2011). In contrast, AZI1’s TMD hydrophobicity index is higher and its targeting to/co‐fractionation with plastids depends on the presence of the PRR (Cecchini *et al*., 2015b; this work). It seems possible that the PRR substitutes for the requirement for a low degree of hydrophobicity within the TMD as the factor for plastid targeting. Interestingly, the PRR has features that are shared with bona fide chloroplastic transit peptides (cTPs). These include a large number of serine, arginine and proline residues, and overall low peptide complexity (targetP, (Emanuelsson *et al*., 2000); (Bruce, 2000; Lee *et al*., 2018)). Thus, AZI1 may use a chimeric targeting mechanism with components shared by signal anchor‐ and cTP‐containing proteins. In this scenario, proteins required for both signal anchor and cTP targeting may also be involved in AZI1 targeting.

Interestingly, an algorithm trained to find apicoplast (plastid analogue in apicomplexan organisms) protein targeting signals, a putative SP+cTP (Zuegge *et al*., 2001), correctly predicts AZI1’s plastid localization (Cecchini *et al*., 2015b). In addition, because transit peptides/presequences and the AZI1 PRR exhibit a high content of positively charged residues (Mackenzie, 2005), the PRR may act as a CPR region (albeit the PRR is longer than the CPRs in signal‐anchored proteins). This would explain our findings that deletion of the AZI1 CPR does not strongly affect AZI1’s targeting.

AZI1’s predicted S‐acylation sites (Ren *et al*., 2008) are conserved in most HyPRP members, suggesting an important role for these residues. However, mutation of these sites did not affect AZI1’s plastid localization. S‐acylation often acts as a mechanism to fine‐tune proteins (re)localization and/or stability (Hemsley, 2015; Daniotti *et al*., 2017). Thus, small differences in AZI1 trafficking dynamics may have been missed in our experiments. Alternatively, the predicted acylation sites (cysteine residues) may be involved in the formation of disulphide bonds and the formation of AZI1‐complexes, as previously suggested (Zhang and Schläppi, 2007; Cecchini *et al*., 2015b).

### MPK3/6 are needed for defense‐related AZI1 plastid targeting and mSAR

As observed after stimulation of R protein‐mediated defenses, MAMP treatment greatly induces the fraction of AZI1 localized to plastids, probably reflecting shared signals and/or components between the SAR and mSAR programs (Mishina and Zeier, 2007; Cecchini *et al*., 2015b). The kinases MPK3 and MPK6 are known shared factors between R‐ and PRR‐induced systemic responses and priming (Beckers *et al*., 2009; Rodriguez *et al*., 2010; Cecchini *et al*., 2019). Remarkably, MPK3 and MPK6 specifically impact AZI1 targeting and are required for the increased accumulation of AZI1 at plastids. Thus, it is possible that MPK3/6 act in systemic defense programs, at least in part, by regulating AZI1’s increased levels in plastids, which in turn may affect the movement of systemic defense priming signals such as AZA or glycerol‐3‐phosphate (Yu *et al*., 2013; Cecchini *et al*., 2015b; Cecchini *et al*., 2019). Moreover, MPK3 and MPK6 can promote plastid‐associated ROS production (Su *et al*., 2018), which play an important role in SAR and could facilitate the generation of AZA in this organelle (Zoeller *et al*., 2012; Wang *et al*., 2014). Considering that MPK3 and MPK6 are activated in response to diverse abiotic stresses (Rodriguez *et al*., 2010; Jalmi and Sinha, 2015), it is also possible that AZI1 is required in plastids during other environmental conditions. We speculate that in these situations, AZI1 could facilitate the movement of other signals, like other plastid oxylipins related to stress resistance (Blée, 2002; Prost *et al*., 2005; Breuers *et al*., 2011).

How might MPK3/6 regulate AZI1’s abundance in plastids? Phosphorylation of cTP residues by STY kinases regulates chloroplast import rate (Martin *et al*., 2006; Lamberti *et al*., 2011). Thus, one idea is that MPK3/6 might impact AZI1 targeting by directly phosphorylating the PRR. In support of this, AZI1’s PRR has several predicted MAPK‐phosphorylation sites and MPK3 can phosphorylate the PRR *in vitro* (Pitzschke *et al*., 2014). Previous work indicated that AZI1 might interact with MPK3 in an *in planta* heterologous system (Pitzschke *et al*., 2014). However, although AZI1 and MPK3/6 partially co‐localized, stable complexes containing AZI1 and MPK3 in *Arabidopsis* were not detectable by co‐immunoprecipitation. Labile and/or short‐lived interactions between AZI1‐MPK3/MPK6 may not permit co‐immunoprecipitation in our conditions. Since MPKs are thought to have many substrates, it is not surprising that stable complexes were not detectable (Bigeard and Hirt, 2018; Rayapuram *et al*., 2018).

### MTs and AZI1 trafficking

Surprisingly, the loss of the N‐terminal TMD causes AZI1 to stably co‐localize with microtubules. AZI1’s PRR and 8CM domain together may directly or indirectly interact with MT filaments, suggesting that AZI1 uses them for intracellular trafficking. Supporting this idea, full length AZI1‐GFP moves along (or very close to) MT bundles. It is unlikely that AZI1‐MT interactions guide AZI1 to plastids, since MT inhibitor treatment did not affect the abundance of AZI1 in plastids. MTs play a key role for the extension of stromules (Erickson *et al*., 2017; Erickson and Schattat, 2018; Kumar *et al*., 2018), plastid structures where AZI1 also resides (Cecchini *et al*., 2015b). Both stromules and AZI1 enrichment in plastids are highly induced with similar kinetics by flg22 ((Caplan *et al*., 2015) and this work). One possibility is that plastid‐anchored AZI1 in close proximity to MTs might help stromules extend to and/or make contact with other structures in cells. It is also possible that the MT network is required for AZI1 to target non‐plastid subcellular sites like plasmodesmata (PD), which was suggested to be required for AZA transport and ultimately systemic resistance (Cecchini *et al*., 2015b). The localization of AZI1 to PD during defense signaling may indicate the importance of AZI1’s trafficking among distinct subcellular compartments for SAR and priming establishment (Lim *et al*., 2016). Since AZI1 is also required for the action of other systemic signals generated in plastids like glycerol‐3‐phosphate, dehydroabietinal and pinene‐monoterpenes (Chaturvedi *et al*., 2012; Yu *et al*., 2013; Riedlmeier *et al*., 2017), it is possible that it also allows small metabolites to reach PDs for simplastic movement. Future work will address these possibilities.

In summary, AZI1 uses a previously undescribed variant of the signal anchor proteins mechanism to target plastids. This targeting and/or stability in the plastid pool of AZI1 is impacted by two key defense associated kinases, MPK3 and MPK6, probably for a tight modulation during defense induction against pathogens and possible other stresses. This could be especially important considering how fundamental plastids are for many types of defense responses (Caplan *et al*., 2008; Grant and Jones, 2009; Nomura *et al*., 2012; Zeier, 2013; de Torres Zabala *et al*., 2015; Cecchini *et al*., 2015a; Medina‐Puche *et al*., 2020). In particular, a timely regulation of the AZI1 pool in plastids could grant an efficient movement of AZA for the establishment of systemic defenses. Additionally, since several HyPRPs contain an N‐terminal bipartite signal, this targeting mechanism could also be functional for all of them. It is also conceivable that other non‐HyPRP plant proteins also use a similar N‐terminal targeting signal. Supporting this idea, several signal‐anchored proteins with high TMD hydrophobicity and predicted to be retained in the ER, were reported to be plastid‐localized (Lee *et al*., 2011).

Collectively, this work strengthens the idea that subcellular re‐localization of defense components is a key aspect of plant immune signaling, especially with regard to the phenomena of priming, in which a well‐placed ambush can be the difference between disease and death.

## Experimental procedures

### Plants

All *Arabidopsis thaliana* plants were 25–28‐day‐old Columbia (Col) ecotype. *mpk3‐1*, *mpk6‐2* and *acd6‐1* were previously described (Lu *et al*., 2003; Liu and Zhang, 2004; Wang *et al*., 2007). *acd6‐1mpk3* double mutant and the Col/Lifeact‐GFP were generated previously (Smertenko *et al*., 2010; Tateda *et al*., 2014). The transgenic plant Col‐*gl1*/GFP‐TUA6 was obtained from ABRC (CS6551) (Ueda *et al*., 1999). *Arabidopsis* plants expressing HA‐tagged AZI1 under dexamethasone (Dex)‐inducible promoter where previously generated (pBAV154:AZI1; (Cecchini *et al*., 2015b)). Plants were grown under 12 h day (8:00 am to 8:00 pm) and 12 h night conditions at 20°C, 200–230 μmol sec^−1^ m^−2^ light at rosette level and 50–70% relative humidity (Jung *et al*., 2009). *Nicotiana benthamiana* were grown at 24°C and with 16 h day light. Plants were grown for 4 weeks before *Agrobacterium tumefaciens*‐mediated transient transformation.

### Vectors and constructs

All primers and vectors used in this study are described in Table S1. The vectors and constructs carrying full length AZI1 as well as the deletion AZI1‐variants II. and VI. were previously described (Cecchini *et al*., 2015b). The AZI1 deletion and point mutation variants as well as the *MPK3* and *MPK6* full coding region were cloned from *A. thaliana* cDNA using PCR primers linked to specific sequences compatible with the TOPO pENTR^®^ and GATEWAY^®^ cloning procedure, and introduced into the plant expression vector pBAV150 (Vinatzer *et al*., 2006). *MPK3* and MPK6 were also introduced into pSITE‐4NA (CD3‐1642/ABRC (Nelson *et al*., 2007)). To generate internal deletion and point mutation constructs the proper primers were used to amplified the fragments to be linked by PCR (Table S2). When required, a start codon was added in the forward primers. All these constructs permitted the expression of the transgenes with C‐terminal GFP or HA‐tag (pBAV150/pBAV154; (Vinatzer *et al*., 2006)) controlled by the Dex‐inducible promoter or with C‐terminal mRFP1 driven by the 35S promoter (pSITE‐4NA). The vector used for the expression of the actin cytoskeleton marker Lifeact‐GFP was previously described (Smertenko *et al*., 2010). The microtubule marker RFP‐TUB6 construct was kindly provided by Dr. Wasteneys (Ambrose *et al*., 2011). All vectors, constructs and primers used in this study are listed in Table S2.

### Subcellular localization

For localization studies, 4‐week‐old *N. benthamiana* leaves were infiltrated with *Agrobacterium tumefaciens* C58C1 or GV3101 strains carrying the different constructs. In order to express fusion proteins from the Dex‐inducible vectors, 20 or 30 μm Dexamethasone (D4902; Sigma‐Aldrich, St. Louis, MO, USA) was infiltrated into leaves 1 day after agroinfiltration. Fractionation and confocal microscopy studies were done 21 h after Dex. *Agrobacterium* harboring different constructs were infiltrated together for co‐expression analysis. *N. benthamiana* leaves were prepared for microscopy as described (Littlejohn *et al*., 2010). A Zeiss LSM710 laser‐scanning confocal microscope (Carl Zeiss Microscopy GmbH, Jena, Germany) was used to capture GFP fluorescence (excitation: 488 nm; emission: 505–530 nm), RFP fluorescence (excitation: 561 nm; emission: 570–620 nm) and plastid autofluorescence (excitation: 633, emission: 650–750 nm). Images were taken using a LD C‐Apochromat 40×/1.1 W Korr objective using a sequential acquisition mode. For time series acquisition, images were taken at low resolution scanning and maximum speed mode. Images were processed using ImageJ (http://rsb.info.nih.gov/ij), ZEN 2012 (Zeiss) and Adobe Photoshop software.

### Quantification of fluorescence

For the analyses of GFP fluorescent intensity of AZI1 and variants in plastids, confocal images were captured using two channels: plastid autofluorescence (blue, 650–750 nm) and GFP fluorescence (green, 505–530 nm) as described above. Using the Fiji software (https://imagej.net/), individual plastids were identified in the blue channel and a spherical region of interest (ROI) was drawn around each plastid such that it incorporated the entire region of autofluorescence. The intensity of the GFP signal within the ROI was measured. Four or more images from independent experiments were analyzed for each construct and 4–5 plastids were quantified per image to calculate the mean plastid GFP fluorescence ± SE. To test the significance across values, one‐way anova followed by Tukey’s HSD post hoc tests were performed. For the analyses of GFP signal that colocalized with RFP‐TUB6, confocal image channels for GFP (green, 505–530 nm) and RFP fluorescence (red, 570–620 nm nm) were used. The ImageJ Colocalization plugin (http://rsb.info.nih.gov/ij/plugins/colocalization.html) was used to generate ROIs from GFP/RFP colocalized signal regions (using thresholds = 50% and ratio = 50%). The intensity of the total GFP signal and within the ROI was measured for each image. Six or more images from independent experiments were analyzed to calculate the percentage of GFP fluorescence ± SE that colocalize with RFP‐TUB6 with respect to total. To test the significance, one‐way anova followed by Tukey’s HSD post hoc tests was performed.

### Fractionation

Microsomal fractions were obtained as described in (Zhang *et al*., 2014). 1 g of *N. benthamiana* leaf tissue expressing the different constructs was used for the fractionation. Plastids were isolated from 1 g of *Arabidopsis* or *N. benthamiana* leaves following the protocol described in (Cecchini *et al*., 2015b). Chlorophyll content was measured by spectrophotometric analysis as described in Lamppa (1995). For *N. benthamiana* transiently expressing AZI1 deletion variants V, VIII, IXc, and X, plastids were isolated from 1 g of leaves following the protocol described in Cecchini *et al*. (2015b) with a slight modification. The total/impure plastids were separated at 4°C on two percoll gradients (80–40%), initially in 2 ml microfuge tubes then again in 4 ml polycarbonate tubes. To enrich for plastid envelopes, purified plastids were frozen in liquid nitrogen to lyse membranes then thawed on ice. Thawed samples were centrifuged at 13 000***g***, the supernatant was collected, then the pellet was resuspended in ice‐cold dH_2_O (with protease inhibitor cocktail) and frozen at −80°C o/n to further lyse membranes. The envelope fraction was again centrifuged at 13 000***g***, the supernatant fraction was collected, then the pellet was resuspended in 1× PBS (1% SDS). Successful fractionation was confirmed by the absence of chlorophyll in the soluble supernatant fractions.

### Western blot analysis and immunoprecipitations

Total proteins or different fractions were separated by SDS‐PAGE. Concentrations of protein in the samples were calculated by Bradford assay (Bradford, 1976). The primary antibodies used for Western blots were: GFP antibody (Covance MMS‐118P, 1:5000), HA antibody (Covance 16B12, 1:1750), HA antibody (Cell Signaling 14031S, 1:1200) , RFP antibody (Agrisera AS15 3028, 1:2000 or 1:3000), MPK3 antibody (Sigma‐Aldrich M8318, 1:3000), MPK6 antibody (Sigma‐Aldrich A7104, 1:3000), phospho‐p44/42 MAPK (Erk1/2) (Thr202/Tyr204) antibody (Cell Signaling Technologies 9101S, 1:1000) (Bartels *et al*., 2009; Beckers *et al*., 2009), H^+^ATPase antibody (Agrisera AS07260, 1:7500), cytosolic FBP antibody (Agrisera AS04 043, 1:3000), BiP2 antibody (Agrisera AS09 481, 1:4000), and rat monoclonal tubulin (yol1/34) antibody (gift from Michael Glotzer, 1:1000). For analysis of native AZI1/EARLI1 proteins an anti‐ AZI1/EARLI1 polyclonal antibody (Zhang and Schläppi, 2007) was used (1:750). The loading buffer for AZI1/EARLI1 Western blot samples does not contain reducing agents as described (Zhang and Schläppi, 2007). Secondary horseradish peroxidase conjugated anti‐rabbit or anti‐mouse antibodies (Thermo Scientific, Rockford, lL, USA) were used at 1:1000. SuperSignal Stable Peroxidase (Thermo Scientific) was used to detect the bands. To quantify by densitometry the Western blot bands and Coomassie Blue staining Gel‐Pro analyzer™ software was used.

For immunoprecipitations, 5 g of leaves of *Arabidopsis* WT or transgenic plants expressing AZI1‐HA was used. Input extracts used for the immunoprecipitation were isolated in extraction buffer (50 mm Tris‐HCL pH8.0, 10 % glycerol, 0.5 % sodium deoxycholate, 1 % Igepal CA‐630 (Sigma‐Aldrich, USA) and complete protease inhibitor cocktail from Roche) as previously described (Cecchini *et al*., 2015b). Proteins were immunoprecipitated using an anti‐HA affinity matrix (rat monoclonal 3F10, Roche). Matrix was washed 4 times with extraction buffer and then resuspended in loading buffer for Western blot analysis.

### flg22 treatment and systemic resistance induction

To analyze AZI1/EARLI1 levels and localization after flg22 peptide treatment, *Arabidopsis* WT and *mpk3* and *mpk6* mutant leaves were infiltrated with 1 µm flg22 or mock (H_2_O). Total or plastid fraction protein samples were obtained after different times post‐infiltration as indicated in Figure legends. Plants were treated soon after lights were turned on in the morning.

To evaluate the local and systemic resistance induced by flg22 (mSAR; Mishina and Zeier, 2007; Cecchini *et al*., 2015b)), 3 lower leaves were infiltrated with 200 nm flg22 or H_2_O (mock). After 2 days local or distal leaves were syringe‐inoculated with virulent *Pseudomonas cannabina* pv *alisalensis* (formerly called *P. syringae* pv. *maculicola* ES4326 (Bull *et al*., 2010)) carrying an empty vector (*Pma*DG3) (OD_600_ = 0.0003) (Guttman and Greenberg, 2001). Bacteria growth was quantified 3 days post infection using 8 leaves from 8 different plants.

### Exogenous application of actin and microtubules inhibitors

Solutions of Latrunculin B (20 µm, Sigma‐Aldrich) or oryzalin (40 µm, Sigma‐Aldrich) inhibitors were directly infiltrated into leaves with a needleless syringe. In *Arabidopsis*, treated leaves were analyzed by microscopy or total and plastid fraction proteins isolated after 3 h. In *N. benthamiana*, discs were immediately cut after leaves’ infiltration and floated in the inhibitor solutions for 16–18 h before the microscopy analysis.

### Statistical analysis

Analyses in this study were done with the software SigmaPlot v11.0 (Systat Software, Inc.). anova (log‐transformed data for bacterial growth curves) followed by the Tukey or Newman‐Keuls (SNK) post hoc test and one‐tailed Student’s *t*‐test were used as indicated in Figure legends.

## AUTHOR CONTRIBUTIONS

NMC and JTG conceived and designed the experiments. NMC, DJS, and SR performed the experiments. JTG, NMC, DJS, and SR analyzed and interpreted data. NMC, DJS and JTG wrote the paper.

## CONFLICT OF INTEREST

The authors declare that they have no conflict of interests.

## Supporting information


**Figure S1**. Quantification of the AZI1:GFP that colocalize with RFP‐TUB6 and anti‐tubulin Western blot of total and plastid fractions from *N. benthamiana* expressing AZI1^Δ2‐25^:GFP (X), AZI1^Δ2‐30^:GFP (XI), or mock‐treated.

**Figure S2.** Flg22 treatment affects the levels of AZI1/EARLI1 and phosphorylation state of MPK3/MPK6 in *mpk3* and *mpk6* mutants.

**Table S1.** A summary of the *in vivo* microscopy and fractionation plastid association data for AZI1 variants.

**Table S2**. Vectors, constructs and primers list used in this study.

**Movie S1.** Live imaging microscopy of AZI1:GFP and RFP‐TUB6 expressing *N. benthamiana*.

## Data Availability

All relevant data can be found within the manuscript and its supporting materials.
