# Supplementary material for: Kinases and protein motifs required for AZI1 plastid localization and trafficking during plant defense induction
Source: Plant J. 2021 Feb 20;105(6):1615–29. doi: 10.1111/tpj.15137 (PMC8048937; doi:10.1111/tpj.15137)
Supplement: Supplementary file 1 — Figure S1. Quantification of the AZI1:GFP that colocalize with RFP‐TUB6 and anti‐tubulin Western blot of total and plastid fractions from N. benthamiana expressing AZI1Δ2‐25:GFP (X), AZI1Δ2‐30:GFP (XI), or mock‐treated. [file TPJ-105-1615-s003.pptx]

## Slide 1
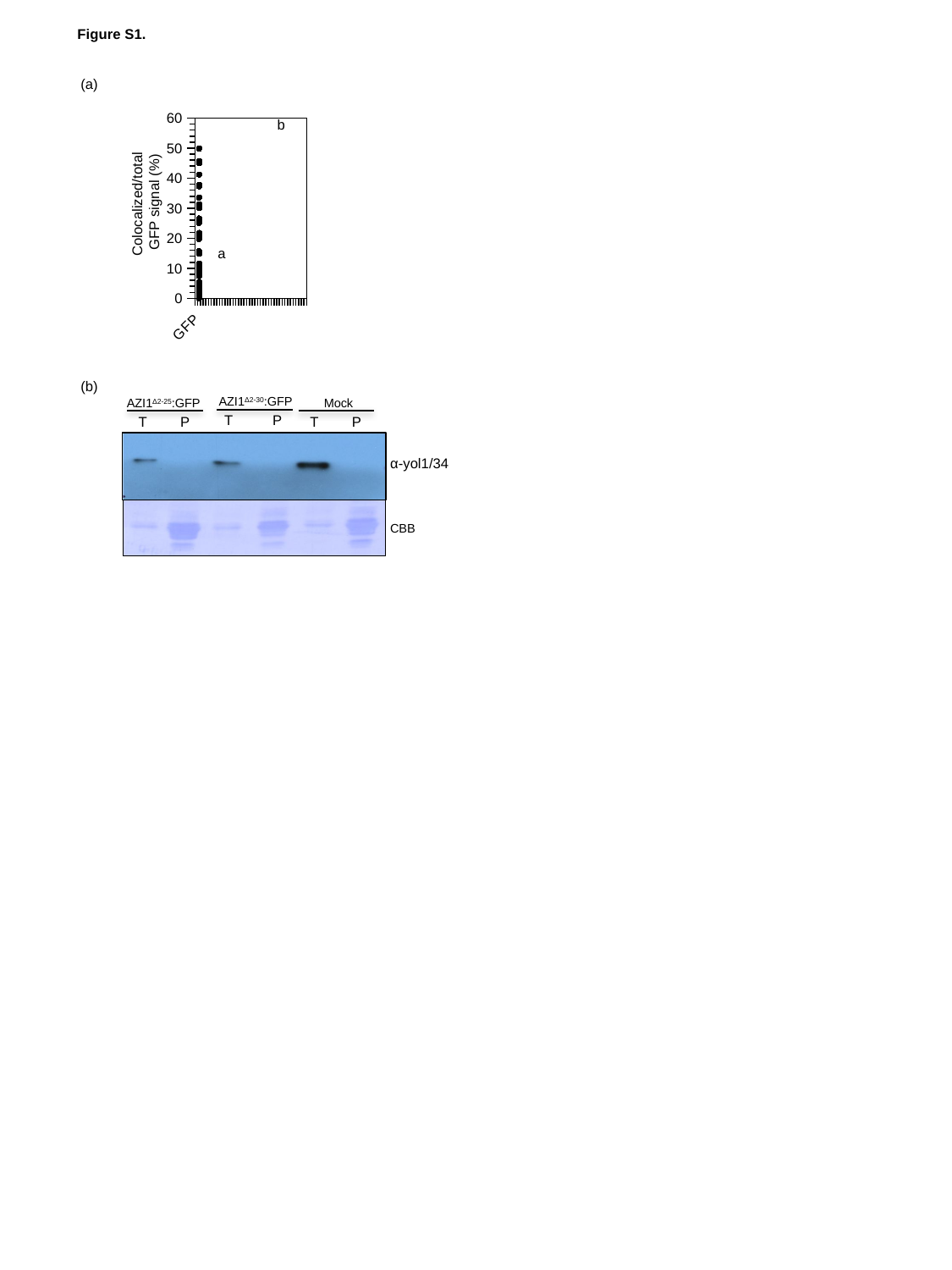

Figure S1.
(a)
### Chart
| Category | | | |
|---|---|---|---|
| GFP | 3.2763923807747353 | 1.195608501567078 | 0.6013881554938844 |
| AZI1:GFP | 16.57752997189816 | 7.110585662450419 | 9.495603298979004 |b
Colocalized/total
 GFP signal (%)
a
(b)
AZI1Δ2-30:GFP
Mock
AZI1Δ2-25:GFP
T
P
T
P
T
P
α-yol1/34
CBB
