# Supplementary material for: Kinases and protein motifs required for AZI1 plastid localization and trafficking during plant defense induction
Source: Plant J. 2021 Feb 20;105(6):1615–29. doi: 10.1111/tpj.15137 (PMC8048937; doi:10.1111/tpj.15137)
Supplement: Supplementary file 2 — Figure S2. Flg22 treatment affects the levels of AZI1/EARLI1 and phosphorylation state of MPK3/MPK6 in mpk3 and mpk6 mutants. [file TPJ-105-1615-s001.pptx]

## Slide 1
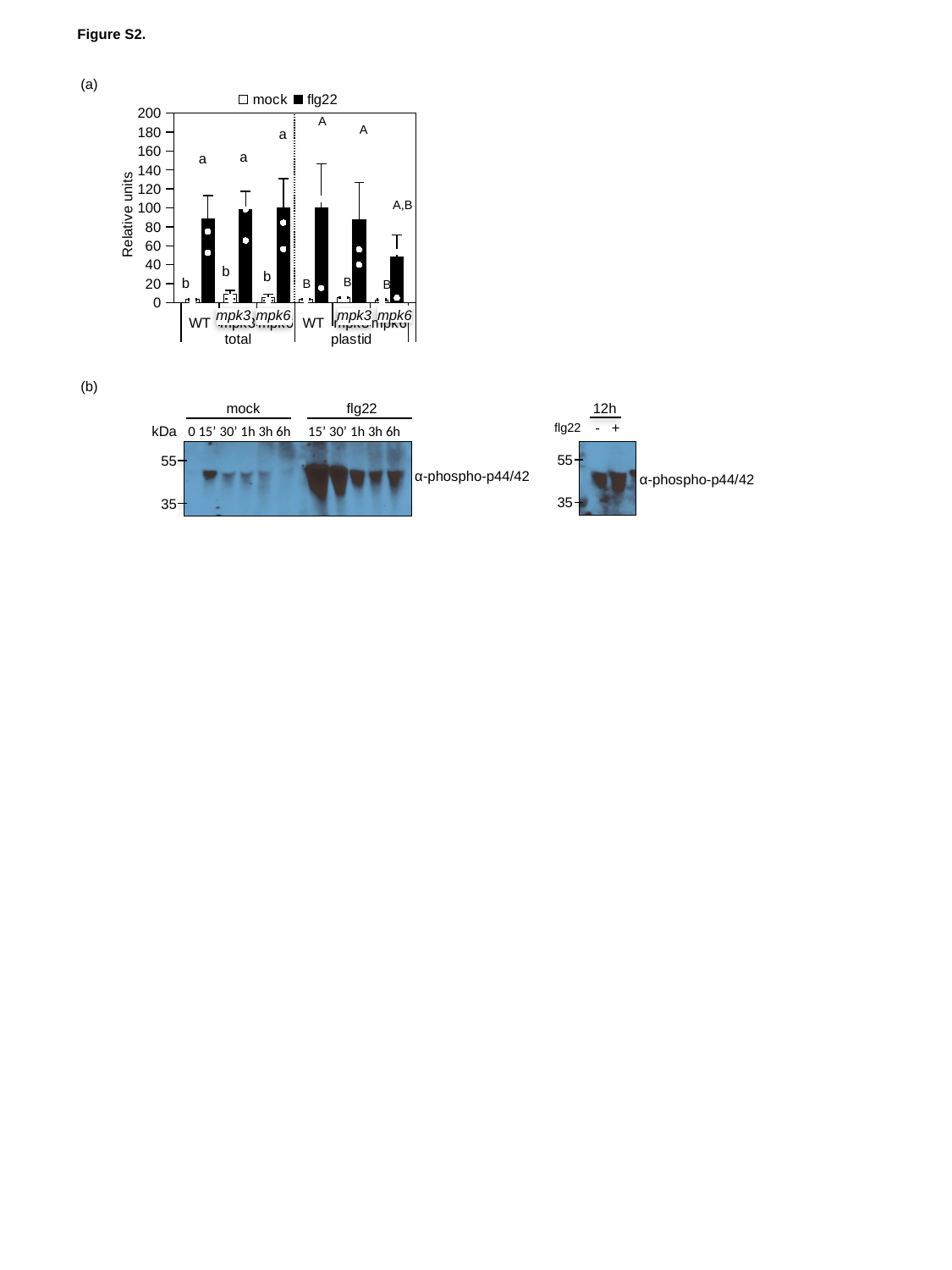

Figure S2.
[unsupported chart]
(a)
A
A
a
a
a
A,B
Relative units
b
b
B
b
B
B
mpk3
mpk6
mpk3
mpk6
(b)
mock
flg22
12h
- +
flg22
kDa
0 15’ 30’ 1h 3h 6h
15’ 30’ 1h 3h 6h
55
55
α-phospho-p44/42
α-phospho-p44/42
35
35
