# Supplementary material for: Kinases and protein motifs required for AZI1 plastid localization and trafficking during plant defense induction
Source: Plant J. 2021 Feb 20;105(6):1615–29. doi: 10.1111/tpj.15137 (PMC8048937; doi:10.1111/tpj.15137)
Supplement: Supplementary file 3 — Table S1. A summary of the in vivo microscopy and fractionation plastid association data for AZI1 variants. [file TPJ-105-1615-s005.docx]

**Table S1.** Summary of the in vivo microscopy and fractionation plastid association data for AZI1 variants.

| **Variants** | **Mutated**  **Regions** | **Plastid localization** | |
| --- | --- | --- | --- |
|  |  | *in vivo*  (microscopy) | fractionation |
| AZI1 | full length | **++** | **+** |
| AZI1^Δ77-161^ | LTP | **++** | **+** |
| AZI1^Δ40-76^ | PRR | **-** | **-** |
| AZI1^Δ32-76^ |  | **-** | **-** |
| AZI1^Δ28-76^ |  | **-** | **-** |
| AZI1^Δ31-37^ | CPR | **+** | **+** |
| AZI1^C28A^ | HD-cysteines | **++** | **+** |
| AZI1^C30A^ |  | **++** | **+** |
| AZI1^C28/30A^ |  | **++** | **+** |
| AZI1^Δ2-25^ | HD | **-**  (+ contact points) | **+** |
| AZI1^Δ2-30^ |  | **-**  (+ contact points) | **+** |

(++), (+), (+/-), (-): indicate level of plastids association for each construct.
