# Supplementary material for: Kinases and protein motifs required for AZI1 plastid localization and trafficking during plant defense induction
Source: Plant J. 2021 Feb 20;105(6):1615–29. doi: 10.1111/tpj.15137 (PMC8048937; doi:10.1111/tpj.15137)
Supplement: Supplementary file 4 — Table S2. Vectors, constructs and primers list used in this study. [file TPJ-105-1615-s002.docx]

**Table S2.** Vectors, constructs and primers list used in this study

| **Vectors/constructs** | | | | |
| --- | --- | --- | --- | --- |
| Plasmid | Parent vector | Description | Antibiotic^a)^ | Reference |
| pBAV150 | TA7001 | Gateway binary plant expression vector (*Dex* promoter, C-terminal HA-tag) | Km^R^/ Cm^R^ /BASTA^R^ | (Vinatzer *et al.*, 2006) |
| pBAV154 | TA7001 | Gateway binary plant expression vector (*Dex* promoter, C-terminal HA-tag) | Km^R^/ Cm^R^ /BASTA^R^ | (Vinatzer *et al.*, 2006) |
| Lifeact-GFP | pMDC43 | CaMV 35S:Lifeact-GFP | Km^R^/Hyg^R^ | (Smertenko *et al.*, 2010) |
| RFP-TUB6 | pCAMBIA1300 | UBQ1::RFP:TUB6 | Km^R^/Hyg^R^ | (Ambrose *et al.*, 2011) |
| pSITE-4NA-MPK3 | pSITE-4NA | 35S::MPK3:mRFP1 (CD-1642) | Sp^R^ | This study |
| pSITE-4NA-MPK6 | pSITE-4NA | 35S-MPK6:mRFP1 (CD-1642) | Sp^R^ | This study |
| pBAV154-AZI1 | pBAV154 | Dex:AZI1:HA in a binary vector | Km^R^ /BASTA^R^ | (Cecchini *et al.*, 2015b) |
| pBAV150-I. AZI1:GFP | pBAV150 | Dex:AZI1:GFP in a binary vector | Km^R^ /BASTA^R^ | (Cecchini *et al.*, 2015b) |
| pBAV150-II. AZI1^Δ77-161^:GFP | pBAV150 | Dex::AZI1-variant:GFP in a binary vector | Km^R^ /BASTA^R^ | (Cecchini *et al.*, 2015b) |
| pBAV150-III. AZI1^Δ38-161^:GFP | pBAV150 | Dex::AZI1-variant:GFP in a binary vector | Km^R^ /BASTA^R^ | This study |
| pBAV150-IV. AZI1^Δ38-161_C28A/C30A^:GFP | pBAV150 | Dex::AZI1-variant:GFP in a binary vector | Km^R^ /BASTA^R^ | This study |
| pBAV150-V. AZI1^Δ40-76^:GFP | pBAV150 | Dex::AZI1-variant:GFP in a binary vector | Km^R^ /BASTA^R^ | This study |
| pBAV150-VI. AZI1^Δ32-76^:GFP | pBAV150 | Dex::AZI1-variant:GFP in a binary vector | Km^R^ /BASTA^R^ | (Cecchini *et al.*, 2015b) |
| pBAV154-VI. AZI1^Δ32-76^:HA | pBAV154 | Dex::AZI1-variant:HA in a binary vector | Km^R^ /BASTA^R^ | This study |
| pBAV150-VII. AZI1^Δ28-76^:GFP | pBAV150 | Dex::AZI1-variant:GFP in a binary vector | Km^R^ /BASTA^R^ | This study |
| pBAV150-VIII. AZI1^Δ31-37^:GFP | pBAV150 | Dex::AZI1-variant:GFP in a binary vector | Km^R^ /BASTA^R^ | This study |
| pBAV150-IX. AZI1^C28A^:GFP | pBAV150 | Dex::AZI1-variant:GFP in a binary vector | Km^R^ /BASTA^R^ | This study |
| pBAV150-IX. AZI1^C28A^:GFP | pBAV150 | Dex::AZI1-variant:GFP in a binary vector | Km^R^ /BASTA^R^ | This study |
| pBAV150-IX. AZI1^C28A/C30A^:GFP | pBAV150 | Dex::AZI1-variant:GFP in a binary vector | Km^R^ /BASTA^R^ | This study |
| pBAV150-X. AZI1^Δ2-25^:GFP | pBAV150 | Dex::AZI1-variant:GFP in a binary vector | Km^R^ /BASTA^R^ | This study |
| pBAV150-XI. AZI1^Δ2-30^:GFP | pBAV150 | Dex::AZI1-variant:GFP in a binary vector | Km^R^ /BASTA^R^ | This study |
| pBAV150-XII. AZI1^Δ2-30/Δ77-161^:GFP | pBAV150 | Dex::AZI1-variant:GFP in a binary vector | Km^R^ /BASTA^R^ | This study |
| pBAV150-XIII. AZI1^Δ2-77^:GFP | pBAV150 | Dex::AZI1-variant:GFP in a binary vector | Km^R^ /BASTA^R^ | This study |
| pBAV150-XIV. AZI1^Δ2-30/Δ103-161^:GFP | pBAV150 | Dex::AZI1-variant:GFP in a binary vector | Km^R^ /BASTA^R^ | This study |
| pBAV150-XV. AZI1^Δ2-52/Δ103-161^:GFP | pBAV150 | Dex::AZI1-variant:GFP in a binary vector | Km^R^ /BASTA^R^ | This study |
| pBAV150-XVI. AZI1^Δ2-52^:GFP | pBAV150 | Dex::AZI1-variant:GFP in a binary vector | Km^R^ /BASTA^R^ | This study |
| **Primers** | | | | |
| Name | | Sequence (5’-3’) | Purpose | |
| Actin qPCR F | | GAGCGGGAAATTGTCAGGGA | qRT-PCR | |
| Actin qPCR R | | GAAACGCTCAGCACCAAT | qRT-PCR | |
| AZI1-cacc-FW | CACCATGGCTTCAAAGAACTCA | | I. to IX. AZI1 variants cloning for constructs | |
| AZI1-nostop-RV | AGCACATTGGAAACCAGATG | | I. / V. to XI. / XIII. / XVI. AZI1 variants cloning for constructs | |
| AZI1_SP+CPR_RV | CTTTGGTTTAGGACTTGGCT | | III. and IV. AZI1 variant cloning for constructs | |
| AZI1 signalP-C-LTP RV | GAGAGCATCAATAGGACAGCTCTTGCAGTTGCA | | VI. AZI1 variant cloning for constructs | |
| AZI1 C-LTP FW | TGCAACTGCAAGAGCTGTCCTATTGATGCTCTC | | VI. AZI1 variant cloning for constructs | |
| SP+CPR---Cterm RV | GAGAGCATCAATAGGACAGCTGACTGGCTTTGG | | V. AZI1 variant cloning for constructs | |
| SP+CPR---AZI1 C-LTP FW | CCAAAGCCAGTCAGCTGTCCTATTGATGCTCTC | | V. AZI1 variant cloning for constructs | |
| SP-CxC-CPR---Cterm RV | GAGAGCATCAATAGGACAGCTATTTGTTGCAAC | | VII. AZI1 variant cloning for constructs | |
| SP-CxC-CPR---AZI1 C-LTP FW | GTTGCAACAAATAGCTGTCCTATTGATGCTCTC | | VII. AZI1 variant cloning for constructs | |
| AZI1w-oCPR_RV | TTGGGACTGGGCAGTTGCAATTTGTTGCA | | VIII. AZI1 variant cloning for constructs | |
| AZI1w-oCPR_FW | ATTGCAACTGCCCAGTCCCAAGTCCTAAG | | VIII. AZI1 variant cloning for constructs | |
| AZI1_C28A_RV | TGGCTTGCAGTTagcATTTGTTGC | | IX. AZI1^C28A^ variant cloning for constructs | |
| AZI1_C28A_FW | GCAACAAATgctAACTGCAAGCCA | | IX. AZI1^C28A^ variant cloning for constructs | |
| AZI1_C30A_RV | TGGCTTagcGTTGCAATTTGTTGC | | IX. AZI1^C30A^ variant cloning for constructs | |
| AZI1_C30A_FW | GCAACAAATTGCAACgctAAGCCA | | IX. AZI1^C30A^ variant cloning for constructs | |
| AZI1_C28A-C30A_RV | TGGCTTagcGTTagcATTTGTTGC | | IX. AZI1^C28A/C30A^ variant cloning for constructs | |
| AZI1_C28A-C30A_FW | GCAACAAATgctAACgctAAGCCA | | IX. AZI1^C28A/C30A^ variant cloning for constructs | |
| AZI1-LTP_FW | caccATGTGTCCTATTGATGCTCTC | | XIII. AZI1 variant cloning for constructs | |
| AZI1-SP_atg_FW | caccatgACAAATTGCAACTGCAAG | | X. AZI1 variant cloning for constructs | |
| caccATG_AZI1 woSP woCxC_FW | caccatgAAGCCAAGTCCTAAACCA | | XI. - XII and XIV. AZI1 variants cloning for constructs | |
| AZI1 N-term -RV | GCTGTTTCCGGATGAACCA | | XII. AZI1 variant cloning for constructs | |
| AZI1_307-Q102_RV | CTGTCCCAACTGGATGTTG | | XIV. and XV. AZI1 variants cloning for constructs | |
| AZI1_157-P53_FW | caccatgCCCCGTCCTTCAGTCCCA | | XV. and XVI. AZI1 variants cloning for constructs | |
| MPK3 FW | CACCATGAACACCGGCGGTGGCCA ATA | | MPK3 cloning for constructs | |
| MPK3RV | ACCGTATGTTGGATTGAGTGC | | MPK3 cloning for constructs | |
| MPK6 FW | CACCAACAATGGACGGTGGTTCAGGTCAAC | | MPK6 cloning for constructs | |
| MPK6RV | TTGCTGATATTCTGGATTGAAAGCAAGC | | MPK6 cloning for constructs | |

a) BASTA^R^, BASTA (glufosinate ammonium) resistance; Cm^R^, chloroamphenicol resistance; Km^R^, kanamycin resistance; Sp^R^, spectinomycin resistance; Rif^R^, rifampicin resistance; Tet^R^, tetracycline resistance.
